# Prediction of Necrotic Core and Hypoxic Zone of Multicellular Spheroids in a Microbioreactor with a U-Shaped Barrier

**DOI:** 10.3390/mi9030094

**Published:** 2018-02-25

**Authors:** Maryam Barisam, Mohammad Said Saidi, Navid Kashaninejad, Nam-Trung Nguyen

**Affiliations:** 1 Department of Mechanical Engineering, Sharif University of Technology, Tehran 11155, Iran; Barisam.m@gmail.com; 2 Queensland Micro- and Nanotechnology Centre, Nathan Campus, Griffith University, 170 Kessels Road, Brisbane QLD 4111, Australia; n.kashaninejad@griffith.edu.au

**Keywords:** microbioreactor, multicellular spheroid with U-shaped barrier, glucose and oxygen concentrations, necrotic and hypoxic regions

## Abstract

Microfluidic devices have been widely used for biological and cellular studies. Microbioreactors for three-dimensional (3D) multicellular spheroid culture are now considered as the next generation in in vitro diagnostic tools. The feasibility of using 3D cell aggregates to form multicellular spheroids in a microbioreactor with U-shaped barriers has been demonstrated experimentally. A barrier array is an alternative to commonly used microwell traps. The present study investigates oxygen and glucose concentration distributions as key parameters in a U-shaped array microbioreactor using finite element simulation. The effect of spheroid diameter, inlet concentration and flow rate of the medium are systematically studied. In all cases, the channel walls are considered to be permeable to oxygen. Necrotic and hypoxic or quiescent regions corresponding to both oxygen and glucose concentration distributions are identified for various conditions. The results show that the entire quiescent and necrotic regions become larger with increasing spheroid diameter and decreasing inlet and wall concentration. The shear stress (0.5–9 mPa) imposed on the spheroid surface by the fluid flow was compared with the critical values to predict possible damage to the cells. Finally, optimum range of medium inlet concentration (0.13–0.2 mM for oxygen and 3–11 mM for glucose) and flow rate (5–20 μL/min) are found to form the largest possible multicellular spheroid (500 μm), without any quiescent and necrotic regions with an acceptable shear stress. The effect of cell-trap types on the oxygen and glucose concentration inside the spheroid was also investigated. The levels of oxygen and glucose concentration for the microwell are much lower than those for the other two traps. The U-shaped barrier created with microposts allows for a continuous flow of culture medium, and so improves the glucose concentration compared to that in the integrated U-shaped barrier. Oxygen concentration for both types of U-shaped barriers is nearly the same. Due to the advantage of using U-shaped barriers to culture multicellular spheroids, the results of this paper can help to choose the experimental and design parameters of the microbioreactor.

## 1. Introduction

Microfluidic systems have been used as practical and powerful tools for cell studies and can replace conventional in vitro systems and even animal models [1,2]. These systems can simulate cellular interactions, gradients and dynamics of tissue microenvironment [3]. They are also widely used for the detection and isolation of tumor cells [4], nanoparticle and drug delivery [5], drug screening [6], chemosensitivity assays [7] and study of tumor microenvironments [8]. On the other hand, multicellular spheroids could serve as a bridge between two-dimensional (2D) monolayer cultures and in vivo models [9,10]. Thus, optimizing microfluidic systems for culturing multicellular spheroids can be very beneficial.

Various methods exist for the formation of spheroids on a chip such as floating on a nonadhesive surface, the hanging-drop method, suspension in spinner flask, acoustic, magnetic or electric force driven methods and mechanical trapping [11,12,13]. Mechanical trapping has been widely used for tumor spheroid generation [14]. This method utilizes barriers such as micropillars or microwells located in the channel to trap the cells and to create multicellular aggregates. A number of researchers [14,15,16] have carried out experiments on devices with U-shaped barriers. Fu et al. [16] concluded from their experiments that this type of device provides the conditions for cell trapping and spheroid generation and culture with continuous perfusion with relatively low shear stress. The present study numerically investigates a microfluidic system with an integrated U-shaped barrier similar to that used in references [14,15,16] and compares its performance with barriers formed by microposts and microwell structure.

Hypoxia and necrosis are essential phenomena in tumors, which may occur in in vitro spheroid culture [17]. Generally, a tumor spheroid has three zones in terms of the availability of oxygen and nutrients, namely necrotic, hypoxic or quiescent and proliferating zones [18]. The availability of oxygen and nutrients improves from the necrotic zone to the proliferating zone. In the necrotic zone, the lack of oxygen and nutrients led to cell death due to starvation. The hypoxic or quiescent zone is important because the lack of oxygen and nutrients causes the cells to secrete growth factors that may lead to angiogenesis [18]. In addition, promoting the expression of hypoxia-inducible genes enhances cancer cell invasion and metastasis [3]. Hypoxia also has a crucial role in tumor progression and subsequent resistance to treatments [19,20]. Therefore, understanding the distributions and the consumption rates of oxygen and nutrients in multicellular spheroids is of great importance for this biological system.

Spheroid diameter is one of the critical parameters that affect the distribution of oxygen and nutrients inside the spheroid. The size of a spheroid can be controlled by cell density and fluid flow during cell seeding process as well as the culture duration. Curcio et al. [21] investigated oxygen, glucose and lactate concentration distribution inside a hepatocyte spheroid both experimentally and numerically (with a simple one-dimensional model). The results indicated that oxygen concentration and cell viability decrease with increasing spheroid diameter. Grimes et al. [9] studied oxygen distribution in tumor spheroids experimentally and analytically. The model was one-dimensional and considered a constant reaction rate. The results showed that hypoxic and necrotic zones become larger by increasing the spheroid diameter. Däster et al. [22] cultured multicellular spheroids with various diameters using the hanging drop method. The authors examined hypoxic and necrotic regions using gene expression analysis and found that these regions appeared for diameters larger than 500 μm. Hu and Li [23] reported a three-dimensional numerical model to study the nutrient concentration distribution inside spheroids. The results indicated that the nutrient concentration reduces with increasing spheroid diameter and decreasing medium flow rate.

Another parameter that affects oxygen concentration distribution is oxygen permeability of the culture chamber or channel wall. Anada et al. [24] experimentally investigated the effect of this parameter on tumor growth. The authors showed that three-dimensional growth of spheroids increases and the probability of hypoxia and necrosis decreases as compared to an impermeable wall. Astolfi et al. [25] experimentally and numerically studied micro-dissected tumor (MDT) in a microfluidic device having some traps. The device has a permeable wall. The results concluded that small spheroid diameter (<420 μm) does not require continuous perfusion. Grist et al. [26] developed a microfluidic device with a main channel for cell culture and three side channels for gas, which can control the oxygen concentration spatially and temporally. Thus, the oxygen concentration inside the spheroid can be tuned, allowing for a parametric study of hypoxia and necrosis.

The trap and aggregate shapes also affect the oxygen and glucose concentration distributions inside the aggregates. We recently examined this parameter numerically [27]. The results showed that the level of oxygen and glucose in a system with U-shaped barrier is higher than that in a system with a microwell. Toroidal and spheroidal shapes of cell aggregates were compared, indicating that the oxygen and glucose concentration levels inside the aggregate depend on the aggregate size and orientation. Spheroids showed the highest risk of hypoxia and necrosis. Thus, a spheroid is chosen for the present study.

This paper aims to numerically investigate oxygen and glucose concentration distributions in multicellular spheroid entrapped in a three-dimensional microfluidic device with an oxygen-permeable wall, an integrated U-shaped barrier or a U-shaped barrier created with microposts or microwell. The effects of all these parameters, namely the spheroid diameter, inlet concentration, media volume flow rate and trap type, on oxygen and glucose concentrations are investigated using finite element approach. Based on glucose and oxygen concentration distributions, hypoxic or quiescent and necrotic zones are identified for each case that can be very useful for designing high-performance microbioreactors.

## 2. Material and Methods

### 2.1. Geometry

**Figure 1:**
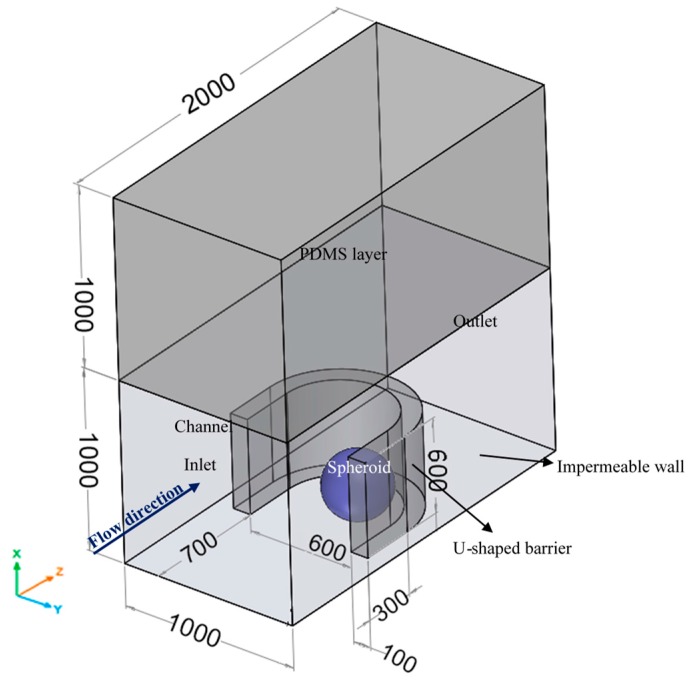
The geometry of an integrated U-shaped microbioreactor used in this study. The system consists of a PDMS U-shaped barrier in a channel with an upper PDMS layer (i.e., the oxygen permeable layer) and a lower glass surface (an oxygen impermeable wall). The spheroid is assumed to be trapped at the boundaries of the U-shaped system. The flow of the culture medium maintains the cellular nutrition and metabolic conditions. All the dimensions are in μm.

Figure 1 illustrates the geometry of the problem. The channel used for cell culture consists of periodic U-shaped barriers in a row for all simulations except those in Section 5.7 as explained later. The spheroid can be formed either on-chip or off-chip [13]. In an on-chip technique, the primary stream carries the seeding cells through the channel. The cells are trapped in the obstacles and formed multicellular spheroids. Another stream washes the remaining cells. Finally, the flow of the culture medium is established to maintain cellular nutrition and metabolic conditions. In an off-chip technique, the spheroid is formed and reached outside the microfluidic system and then transferred to and cultured in the microbioreactor. Our numerical simulations are based on the off-chip technique.

We assumed that the upper channel walls and U-shaped barriers are made of the commonly used elastomer polydimethylsiloxane (PDMS), which is permeable to oxygen. The channel lower wall is made of PDMS but placed on an impermeable surface like glass surface. Furthermore, assuming other obstacles in a row, both surfaces are considered to be symmetrical. Table 1 lists the geometric parameters of the model.

### 2.2. Governing Equations and Boundary Conditions 

In this model, the medium flow through the channel and around the spheroid was considered as a steady, incompressible, three-dimensional and laminar flow. The medium has hydrodynamic properties similar to those of water at 37 °C. The fluid flow is governed by the continuity and Navier–Stokes equations [28] (Equations (1) and (2)).

(1) $$\nabla \cdot \vec{u}=0$$

(2) $$\rho \left(\vec{u}\cdot \vec{\nabla }\right)\;\vec{u}=-\vec{\nabla }p+\mu \nabla^{2}\vec{u}$$

where $\vec{u}$, p, ρ and μ are the medium velocity, pressure, density and viscosity, respectively. 

Figure 1 shows the inlet and outlet of the channel. Considering the geometrical dimensions and the mean free path of water molecules, the no-slip boundary condition for the walls is applied to all the walls including the surface of the spheroid. Fully-developed velocity based on the flow rate (*Q* = 5,10,15,20 μL/min) and zero-pressure are imposed at the inlet and outlet, respectively. The values of simulation parameters are presented in Table 2.

There are generally two different approaches for modelling a multicellular aggregate, viz. discrete approach and continuum approach. Each approach has its own advantages and limitations. The discrete or cell-based approach is computationally complex and is suitable for modeling the dynamic process of tumor growth, as cell interaction plays an important role. The continuum approach is a macroscopic (top-down) model based on a spatially-averaged standpoint. This approach is a suitable for considering epigenetic variation such as environmental variables. Since the main aim of the present study is to determine the oxygen and nutrient distributions available from the external environment of the spheroid, we adopted the continuum approach, which can be coupled with the floand concentration distribution around the spheroid. In addition, our model is based on off-chip technique. Therefore, dynamic tumor growth plays a marginal role. Accordingly, we take into account the effect of cells by considering the Michaelis–Menten reaction terms for both oxygen and glucose.

To investigate the distribution of oxygen concentration, convection–diffusion equation in the medium (Equation [3] without the transient and reaction terms), reaction–diffusion equation in the spheroid (Equation [3] without transient and convection terms) and diffusion equation in the barrier and the upper PDMS layer (Equation [3] without transient, convection and reaction terms) were numerically solved. Because of the no-flow condition inside the spheroid and the PDMS material, convection term for these domains are set to zero. A reaction rate based on Michaelis–Menten model is considered inside the spheroid to model the oxygen consumption rate of the cells. This model has been used in previous studies [21,23,25,29,30].

(3) $$\frac{\partial c}{\partial t}+\vec{u}\cdot \vec{\nabla }c=\vec{\nabla }\cdot (K\vec{\nabla }c)-R$$

(4) $$R=\frac{V_{max}c}{c+K_{m}}$$

where *K* is the diffusion coefficient of oxygen, *c* is concentration, *t* is time, and *R* is the reaction term. $V_{max}$ is the maximum reaction rate and $K_{m}$ is the Michaelis constant.

The channel inlet has a fixed concentration ($c_{0,O_{2}}$ = 0.02–0.2 mM) for dissolved oxygen in the medium. Constant concentration is applied on the upper surface of the PDMS layer ($c_{upper\;wall}=S_{O_{2}–PDMS\;vs.\;H_{2}O}\times c_{0,O_{2}}$ to consider concentration jump) to model the diffusion of oxygen from the surrounding air into the culture chamber. The lower surface of the channel has no-flux condition because of the impermeable surface. As mentioned previously, symmetry boundary conditions are applied at the side surfaces (all fluxes across these boundaries are considered to be zero). At the interfaces (the surface between culture medium and the spheroid or the surface between culture medium and the PDMS material), equal mass fluxes ($J_{O_{2},\;aggregate}=\;J_{O_{2},\;medium}\;\mathrm{and}\;J_{O_{2},\;PDMS}=\;J_{O_{2},\;medium}$, *J* is diffusion mass flux) and concentration jump due to the difference in the partition coefficients ($c_{O_{2},\;aggregate}=S_{O_{2}–cancerous\;tissue\;vs.\;H_{2}O}\times c_{O_{2},\;medium}$ and $c_{O_{2},\;PDMS}=S_{O_{2}–PDMS\;vs.\;H_{2}O}\times c_{O_{2},\;medium}$) are considered.

The convection–diffusion equation and the reaction–diffusion equation are also used to describe the glucose concentration in the medium channel and the spheroid, respectively. Since PDMS is impermeable to glucose, no equation is solved for the glucose in this domain. In all results except those of Section 5.8, steady-state form of Equation (3) is solved. The channel inlet is set with constant concentration ($c_{0,glucose}$ = 0.7–11 mM). Similarly, Michaeles–Menten reaction is applied to the spheroid to take into account the effect of cellular consumption rate. Top and bottom walls and the side walls are conditioned with no-flux conditions and symmetry boundary conditions, respectively. Equal fluxes and concentration is applied to the interface of the spheroid and the channel ($J_{glucose,\;aggregate}=\;J_{O_{2},\;medium}$ and $c_{glucose,\;aggregate}=S_{glucose–cancerous\;tissue\;vs.\;H_{2}O}\times c_{glucose,\;medium}$).

Once the concentrations of glucose and oxygen reach below the amount considered for necrosis, these values are set to be constant.

All domains are meshed with tetrahedral grids. Mesh refinement is applied near the interfaces and boundaries. Finite element method is used to find approximate solutions for nonlinear and linear equations of this study. Residual values less than 10^−6^ for continuity and momentum equations and 10^−3^ for convection–diffusion equations are used as convergence criteria.

## 3. Convergence and Grid Independence Study

**Figure 2:**
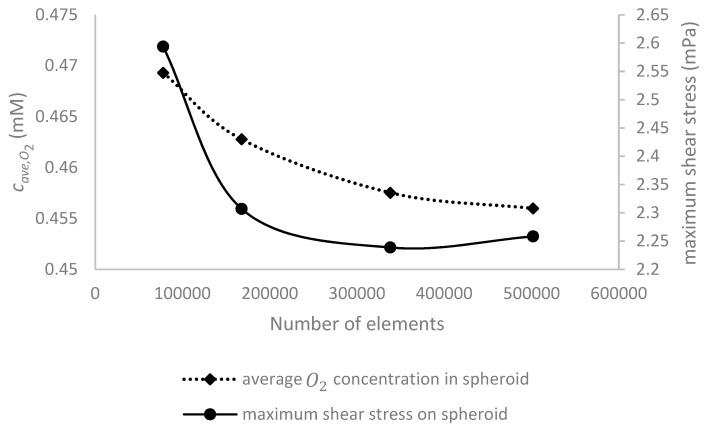
Finite element convergence and grid independence verification on the simulated results. The average oxygen concentration inside the spheroid (the left vertical axis) and maximum shear stress on its surface (the right vertical axis) are plotted as a function of the number of computational elements. The simulations were performed with *D* = 500 μm, *Q* = 5 μL/min and $c_{0,O_{2}}=0.2\;\mathrm{mM}$.

To show the independency of the results from the number of computational elements, for different grids, maximum shear stress on the surface of the spheroid and average oxygen concentration in the whole volume of the spheroid were measured for *D* = 500 μm, *Q* = 5 μL/min and $c_{0,O_{2}}=0.2\;\mathrm{mM}$. Figure 2 shows the averaged oxygen concentration in the spheroid and the maximum shear stress on the spheroid as functions of number of elements. 

We observed that increasing the number of elements makes the data to converge to an approximately constant value. With a total number of cells of 338,220, the error is less than 1% compared to that with the finer mesh.

## 4. Validation

To verify the simulation results, our numerical results were compared with both analytical and experimental data and presented in our previous work [27]. We compared numerical and analytical results of the distribution of oxygen concentration inside a spheroid with a constant surface concentration and consumption rate. Also, for a fixed spheroid surface concentration, the radius with a specific partial pressure of oxygen (the hypoxia boundary) was obtained as a function of the spheroid radius and compared with experimental data. The results showed an excellent agreement. 

**Figure 3:**
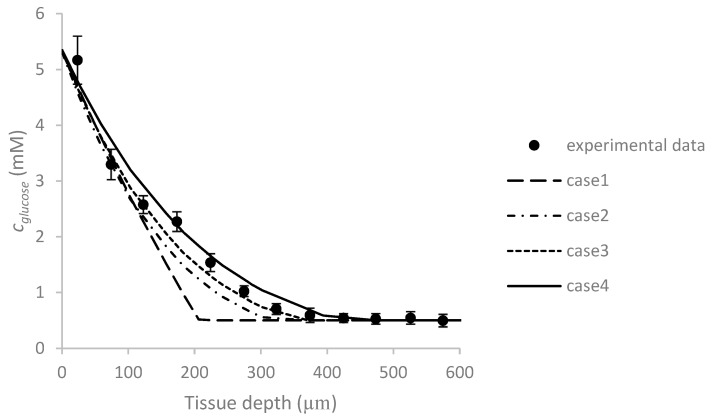
The comparison between the glucose concentration distribution inside tumor aggregate as a function of tissue depth obtained from present numerical results (case 1: $V_{max}=0.01076\;\mathrm{mM}/s$ and $K_{m}=0.04\;\mathrm{mM}$, case 2: $V_{max}=0.05773\;\mathrm{mM}/s$ and $K_{m}=2.6\;\mathrm{mM}$, case 3: $V_{max}=0.05206\;\mathrm{mM}/s\;$and $K_{m}=3.1\;\mathrm{mM}$ and case 4:$\;V_{max}=0.03596\;\mathrm{mM}/s\;$and $K_{m}=2.9\;\mathrm{mM}$) and experimental data conducted by Panteli and Forbes [31]. For both experiment and simulation, the flow rate and initial glucose concentration are *Q* = 3 μL/min and $c_{0,glucose}=$ 5.5 mM, respectively.

The aforementioned cases entailed static conditions, without a flow through the system. To demonstrate the validity of the results even under dynamic conditions, a comparison is performed between numerical and experimental data derived from a perfusion-based system. Panteli and Forbes [31] cultured cancerous LS174T colon adenocarcinoma cells as a tumor aggregate in a microfluidic T-shaped system. The authors presented glucose concentration profile inside the aggregate. The medium flow rate through the main channel and the glucose inlet concentration were 3 μL/min and 5.5 mM, respectively. Here, the numerical simulation is performed under different Michaelis–Menten constants and compared with the experimental ones, Figure 3. The constants in case 1 is used for glucose uptake in ovarian and prostate multidissected tumors (MDT) in [25] ($V_{max}=0.01076\;\mathrm{mM}/s$ and $K_{m}=0.04\;\mathrm{mM}$) and in case 2 and 3 are obtained for glucose consumption in the breast cancer cells (case 2: $V_{max}=0.05773\;\mathrm{mM}/s$ and $K_{m}=2.6\;\mathrm{mM}$ and case 3: $V_{max}=0.05206\;\mathrm{mM}/s$ and $K_{m}=3.1\;\mathrm{mM}$) and case 4 is obtained for the RA (retinoic acid)-treated breast cancer cells [32] ($V_{max}=0.03596\;\mathrm{mM}/s$ and $K_{m}=2.9\;\mathrm{mM}$). Considering the experimental data, we assumed that necrosis occurred at 0.5 mM. According to Figure 3, our numerical results can predict the trend of the profile reasonably well. However, it is necessary to choose the best consumption constants based on the cell type. 

## 5. Results and Discussion

### 5.1. Effect of Spheroid Diameter on Oxygen and Glucose Concentration Distribution 

**Figure 4:**
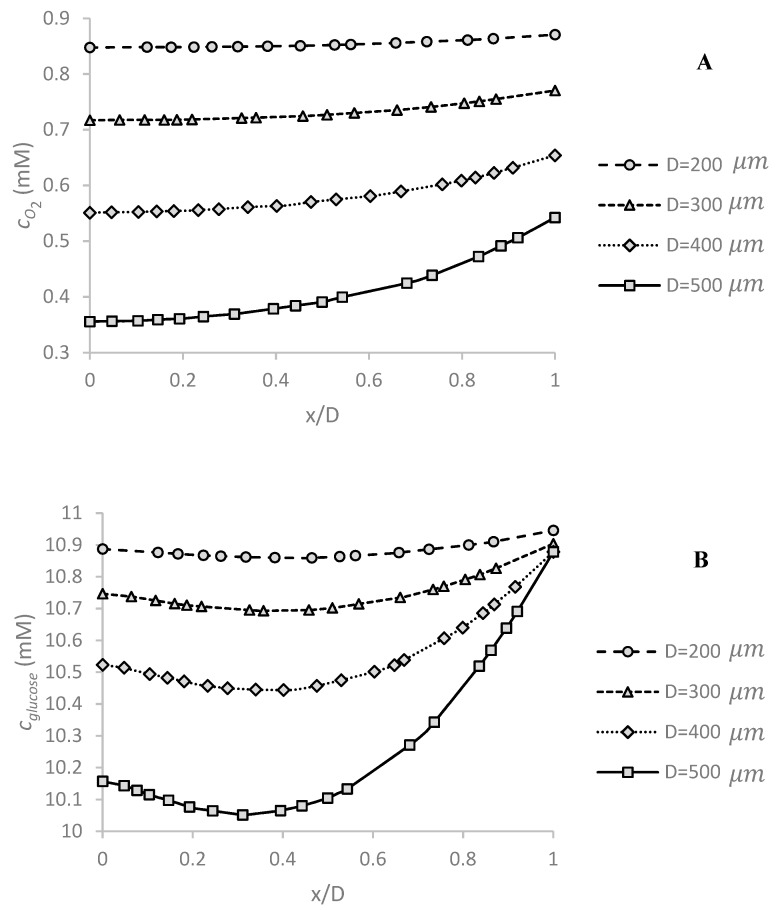
The effect of spheroid diameter *D* on (**A**) oxygen and (**B**) glucose concentration profiles in the center of spheroid along *x*-axis (function of dimensionless *x* position: *x*/*D*). The maximum oxygen and glucose concentration difference in the spheroid increases, respectively from ~3% to ~52% and from ~1% to ~8% as the spheroid diameter increases from 200 μm to 500 μm. The simulations are done under *Q* = 5 μL/min, $c_{0,O_{2}}=0.2\;\mathrm{mM}$ and $c_{0,glucose}=11\;\mathrm{mM}$.

Spheroid diameter is a critical parameter for examining the possibility of hypoxia or necrosis. As the spheroid diameter increases, diffusion of oxygen and glucose to inner regions becomes more difficult. Figure 4A shows the effect of this parameter on oxygen concentration distribution in the center of the spheroid along *x*-axis as shown in Figure 1 under $c_{0,O_{2}}=0.2\;\mathrm{mM}$ and *Q* = 5 μL/min. Since the boundary conditions are asymmetrical, the obtained concentration profile is completely asymmetric to the center of the spheroid. The minimum concentration is at the bottom, because of the low convection term in the spheroid surrounded in this region. The maximum mismatch in oxygen concentration in the spheroid increases from ~3% to ~52% as the spheroid diameter increases from 200 μm to 500 μm. As expected, oxygen concentration significantly decreases with increasing spheroid diameter.

Figure 4B shows the effect of the spheroid diameter on the glucose concentration profile along the center line of the spheroid under the stated conditions. Despite the oxygen concentration, glucose minimum concentration occurs at the higher location than the bottom surface of the channel, because of the low diffusion coefficient of glucose in the tissue ($K_{glucose–cancerous\;tissue}\approx 0.14\;K_{O_{2}–cancerous\;tissue}$). This trend was observed in the study of Astolfi et al. [25]. By taking away the location of the minimum concentration, the glucose concentration increases. Glucose concentration decreases in the spheroid nonlinearly as the spheroid diameter increases. The maximum concentration difference in the spheroid changes from ~1% to ~8% by increasing the spheroid diameter from 200 μm to 500 μm. The effect of diameter on glucose concentration is less significant than oxygen concentration in all spheroids. The main reason is the difference of their reaction rate.

### 5.2. Effect of Volume Flow Rate of Medium on Oxygen and Glucose Concentration Profile

**Figure 5:**
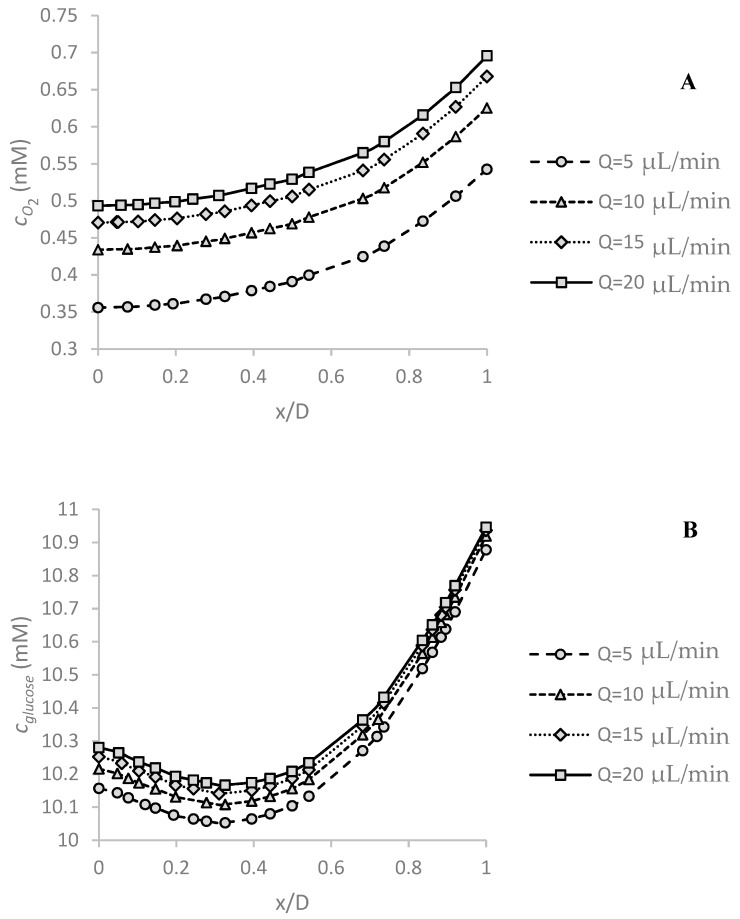
The effect of medium volume flow rate *Q* on (**A**) oxygen and (**B**) glucose concentration profiles in the center of spheroid along *x*-axis (defined based on the dimensionless *x* position: *x*/*D*). The effect of changing the flow rate is more pronounced on the oxygen concentration compared to that on the glucose. When the medium flow rate increases by 300%, the maximum concentrations of oxygen and glucose increase by 38% and 1%, respectively. The simulations were carried out for *D* = 500 μm, $c_{0,O_{2}}=0.2\;\mathrm{mM}$ and $c_{0,glucose}=11\;\mathrm{mM}$.

Figure 5A,B show the effect of medium volume flow rate on oxygen and glucose concentration distribution, respectively. The simulations were carried out under $c_{0,O_{2}}=0.2\;\mathrm{mM}$, $c_{0,glucose}=11\;\mathrm{mM}$ and *D* = 500 μm corresponding to the largest spheroid and the highest concentration. By increasing the flow velocity, more oxygen is available in the vicinity of the spheroid, leading to an increase of oxygen concentration. This enhancement is nonlinear and reduces as the flow rate increases.

The volume flow rate also affects the glucose concentration. The glucose concentration enhanced slightly by increasing the medium flow rate, Figure 5B. A 300% increase in medium flow rate leads to an enhancement of maximum concentration of almost 38% for oxygen and of almost 1% for glucose. Thus, the effect of medium flow rate on oxygen concentration is more significant than glucose concentration. Glucose is a large molecule with low diffusion, and therefore is less affected by the fluid flow.

### 5.3. Effect of Medium Flow Rate and Spheroid Diameter on Maximum Shear Stress Applied to the Spheroid Surface

**Figure 6:**
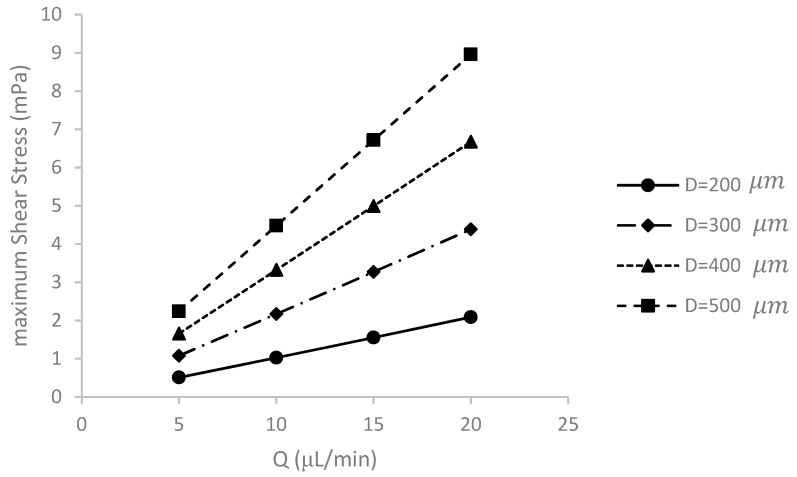
The effect of variation of the culture medium volume flow rate *Q* and spheroid diameter *D* on the maximum shear stress applied to the surface of the spheroid. In all cases, the maximum shear stress is well below the shear stress threshold of 25 mPa for cancer cells.

One of the most important parameters in designing microfluidic systems suitable for cell culture is the shear stress acting on the cells, as it can affect the function, differentiation or proliferation of some cells [33]. In this section, the maximum shear stress on the spheroid under different conditions is modeled and discussed, Figure 6.

Increasing the volumetric flow rate increases the maximum shear stress imposed on the spheroid surface. Noticeably, increasing the velocity increases the difference in the velocity of the fluid flow layers due to the no-slip condition, and hence increases the shear rate and subsequently the shear stress. 

By increasing the diameter of the spheroid, the deviation of streamlines becomes more apparent. The upper surface of the spheroid is exposed directly to the flow and experiences a large shear stress.

In all cases, the maximum shear stress are lower than the threshold of 25 mPa, which is the maximum shear stress that cancer cells endure under physiological condition [34]. Thus, the design under investigation is suitable for cell culture.

### 5.4. Effect of the Inlet Concentration $c_{0}$ on Oxygen and Glucose Concentration Distributions

**Figure 7:**
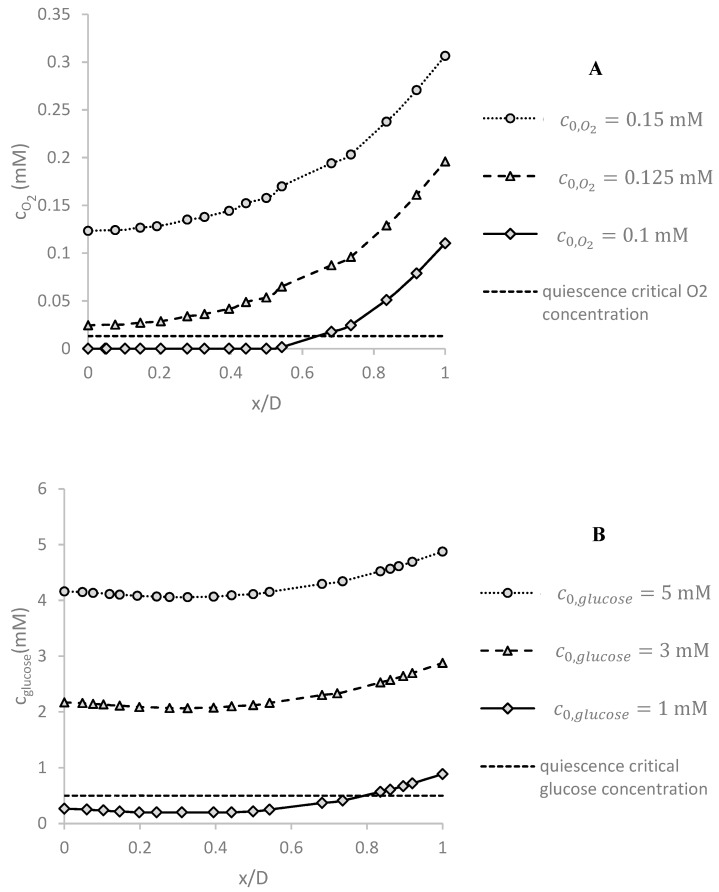
The effect of inlet concentrations of glucose ($c_{0,glucose}$) and oxygen ($c_{0,O_{2}}$ on (**A**) glucose and (**B**) oxygen concentration profile in the center of spheroid along *x*-axis (based on the dimensionless x position: *x*/*D*), respectively. Hypoxia or quiescence is defined when oxygen or glucose concentration is less than the critical value of $c_{O_{2}}=0.01322\;\mathrm{mM}$ and $c_{glucose}=0.5\;\mathrm{mM}$. If the inlet concentrations of glucose and oxygen are equal or less than 1 mM and 0.1 mM, respectively, some regions of the spheroid are in necrosis and hypoxic conditions. The simulations were done for *Q* = 5 μL/min and *D* = 500 μm.

As mentioned before, $c_{0,O_{2}}$ is the concentration of $O_{2}$ at the inlet and $1/\left(S_{O_{2}–PDMS\;vs.\;H_{2}O}\right)$ is the concentration of the top surface of the PDMS layer. By decreasing $c_{0,O_{2}}$, the level of oxygen inside the spheroid decreases significantly as shown in Figure 7A. The simulations are done at *Q* = 5 μL/min and *D* = 500 μm. For $c_{0,O_{2}}=0.1\;\mathrm{mM}$ in the region of spheroid, the hypoxia (O_2_ partial pressure is below 10 mmHg [9,35] equivalent to $c_{O_{2}}=0.01322\;\mathrm{mM}$) and even necrosis (O_2_ partial pressure near 0 mmHg [6] equivalent to $c_{O_{2}}=0\;\mathrm{mM}$) is observed. Therefore, it is necessary to keep $c_{0,O_{2}}$ as high as possible. 

Figure 7B shows the effect of glucose inlet concentration on the profile of glucose concentration distribution under *Q* = 5 μL/min and *D* = 500 μm. For low glucose concentration $c_{0,glucose}$, the glucose level in some regions falls below the critical level and can damage the cells as explained in more details in the next section.

### 5.5. Quiescent and Necrotic Zones Based on Oxygen Concentration

**Figure 8:**
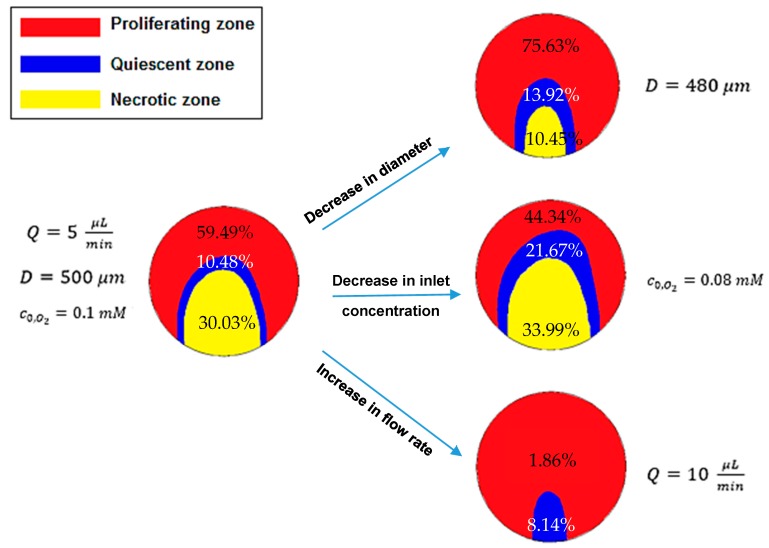
The effects of the diameter *D*, the inlet concentration of oxygen $c_{0,O_{2}}$ and the flow rate *Q* on the proliferating, quiescent and necrotic zones (red, blue and yellow, respectively) in the middle *x*–*z* plane of the spheroid based on oxygen concentration distribution. (The percentage of each region area to the total area of that cross section is reported on that region).

As explained above, the hypoxic or quiescent region and necrotic zone are identified by oxygen partial pressure of 10 mmHg and 0 mmHg, respectively. In this section, the effect of spheroid diameter, medium flow rate and $c_{0,O_{2}}$ on the approximate regions in the middle *x*–*z* plane of the spheroid, equivalent to those pressures is examined. The results are shown in Figure 8. Left regions (hypoxic and necrotic regions) are obtained under *Q* = 5 μL/min, *D* = 500 μm and $c_{0,O_{2}}=0.1\;\mathrm{mM}$ and right regions achieved when the spheroid diameter decreases to 480 μm, the flow rate increases to 10 μL/min and $c_{0,O_{2}}$ reduces to 0.08 mM. The reported percentages are the ratio of the area of each region to the total area of that cross section.

Figure 8 indicates that necrotic and quiescent regions become smaller by increasing $c_{0,O_{2}}$ and the medium flow rate and by decreasing the spheroid diameter. Therefore, both regions disappear with the high oxygen concentration, high medium flow rate or for small spheroids. Actually, with high outer oxygen partial pressure, the concentration inside the spheroid is expected to become higher. A higher medium flow rate means more dissolved oxygen. Small spheroids need less oxygen. Thus, perfusion of oxygen in the spheroids depth is higher, which is consistent with the results shown in Figure 8 and reported in reference [36], which concluded that hypoxic and necrotic zones typically exist in the spheroids with a diameter larger than 200 μm and 500 μm, respectively. 

Particularly, a 4% decrease in the spheroid diameter resulted in almost a 39.8% decrease in total necrotic and quiescent regions, so the change in total quiescent and necrotic areas per change in the spheroid diameter is about 9.95. Similarly, a 20% decrease in $c_{0,O_{2}}$ and a 100% increase in the culture medium flow rate resulted in 37.4% increase and 79.9% reduction in the total area of necrosis and quiescence, respectively. As a consequence, the changes in total quiescent and necrotic area per the changes in $c_{0,O_{2}}$ and the medium flow rate are about 1.87 and 0.8, respectively. Hence, the spheroid diameter has the most and, the medium flow has the least impact on these regions. These analyses are based on the area of mentioned regions in the middle *x*–*z* plane of the spheroid, not on the basis of the volume of these regions.

One of the remarkable results is that these regions do not have the spherical shapes. It is due to the asymmetric oxygen concentration distribution around the multicellular spheroid.

### 5.6. Quiescent and Necrotic Zones Based on Glucose Concentration

**Figure 9:**
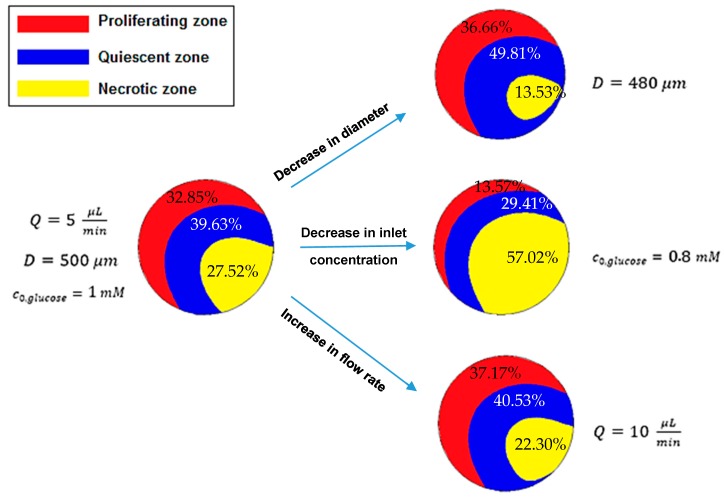
The effects of the diameter *D*, the inlet concentration of oxygen $c_{0,glucose}$ and the flow rate *Q* on the proliferating, quiescent and necrotic zones (red, blue and yellow, respectively) in the middle *x*–*z* plane of the spheroid based on glucose concentration distribution. The percentage of each region area to the total area of that cross section is reported on that region.

This section examines the effect of glucose concentration on areas of deficiency (quiescence) and areas of necrosis. Similar to the previous section, we investigated the effect of inlet concentration, spheroid diameter and medium flow rate on these regions. The border of these regions are identified by $c_{glucose}=0.2\;\mathrm{and}\;0.5\;\mathrm{mM}$, respectively (because of lack of information the data for the intervertebral disc (IVD) [37] used in this study). Figure 9 shows the simulation results. As defined in the previous section, the percentages show the ratio of the area of each region to the total area of that cross section.

An increasing spheroid diameter, decreasing inlet concentration and medium flow rate lead to the growth of the quiescent and necrotic region. Small spheroids and high inlet concentration and a medium flow rate reduce these zones.

Similar to the previous section, a 4% decrease in the spheroid diameter, a 20% decrease in $c_{0,glucose}$ and a 100% increase in the culture medium flow rate resulted in almost a 5.7% decrease, a 28.7% increase and 6.4% decrease in the total area of necrosis and quiescence, respectively. The changes in total quiescent and necrotic areas per the changes in the spheroid diameter, $c_{0,glucose}$ and the medium flow rate are about 1.42, 1.43 and 0.06, respectively. As a result, the spheroid diameter and the glucose concentration $c_{0,glucose}$ have almost an equal effect on these regions. The medium flow has the least impact. The difference made by glucose is less than oxygen. These analyses are based on the area of mentioned regions in the middle *x*–*z* plane of the spheroid too.

The quiescent and necrotic regions due to deficiency of oxygen are located at the lower part of the spheroid. The quiescent and necrotic zones due to deficiency of glucose tend to locate at the back of the spheroid, where it is not exposed to direct flow. The reason is that the barrier is permeable to oxygen and not to glucose. This feature increases the risk of damage to cells due to lack of glucose. These regions are also nonspherical due to asymmetric glucose concentration distribution around the spheroid. 

### 5.7. Comparison of the Different Trap Types

**Figure 10:**
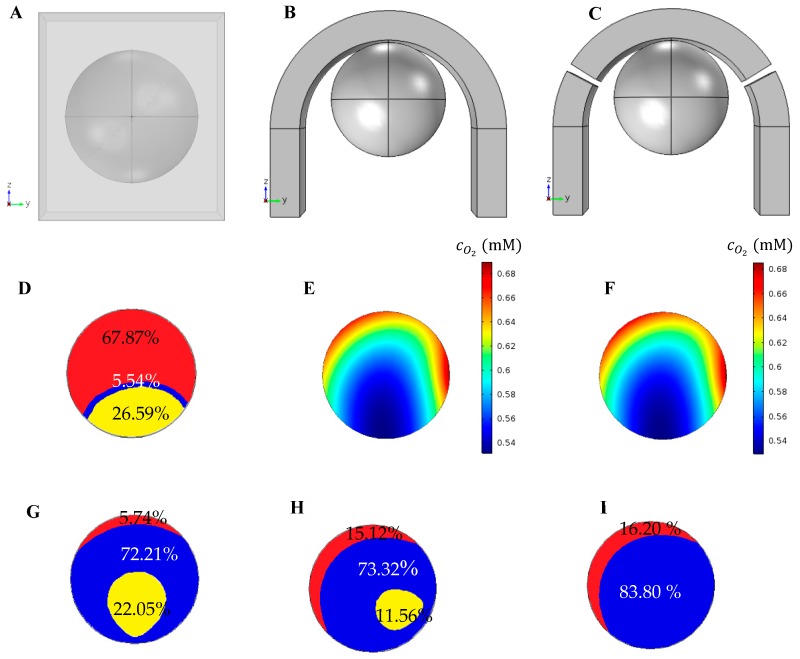

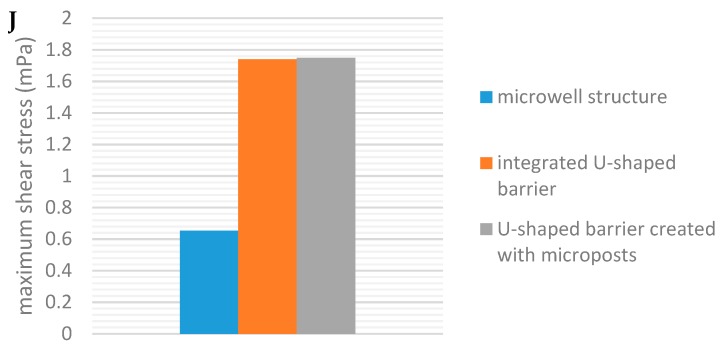
Comparison of the different traps, i.e., (**A**) microwell structure; (**B**) integrated U-shaped barrier and (**C**) U-shaped barrier created with microposts, on the oxygen (**D**–**F**) and glucose (**G**–**I**) concentration distribution inside the spheroid and maximum shear stress on its surface (**J**,**E**) the proliferating, quiescent and necrotic zones due to oxygen deficiency in the middle *x*–*z* plane of the spheroid in the microwell, (**F**,**G**) oxygen concentration distribution in the middle *x*–*z* plane of the spheroid in the integrated U-shaped barrier and U-shaped barrier created with microposts, respectively, (**H**–**J**) the proliferating, quiescent and necrotic zones due to glucose deficiency in the middle *x*–*z* plane of the spheroid in the microwell, U-shaped barrier and U-shaped barrier created with microposts, respectively (the simulations are done under *D* = 400 μm, *Q* = 5 μL/min, $c_{0,O_{2}}=0.2\;\mathrm{mM}$ and $c_{0,glucose}=0.7\;\mathrm{mM}$).

This section investigates the effect of a trap type with a different design on the distribution of oxygen and glucose concentration in the spheroid and the amount of shear stress on its surface. The general geometry is similar to the one shown in Figure 1. Each case considers one of the traps depicted in Figure 10A–C. In Figure 10A, the spheroid is in a rectangular microwell with the same trap length, area and height as the one presented in [27]. Figure 10B shows again the U-shaped barrier used for all simulations in previous Sections. The U-shaped barrier in Figure 10C is created with some microposts and so, two gaps of 2 degrees are considered at angles of 30 and 150 degrees that allow the culture medium flow through. The similar design was used by Ong et al. [38]. All three traps have an equal height (400 µm). The simulations are performed under *D* = 400 μm, *Q* = 5 μL/min, $c_{0,O_{2}}=0.2\;\mathrm{mM}$ and $c_{0,glucose}=0.7\;\mathrm{mM}$. 

Figure 10D–F show that the concentration of oxygen in the U-shaped barrier is far higher than that in the microwell. Inside the spheroid in a microwell, the quiescence and necrosis are created in the predefined conditions (Figure 10D). Such areas are not created for two other traps, and the oxygen levels there were also very high (Figure 10E,F). The oxygen concentrations inside the spheroid in the integrated U-shaped barrier and the one created with microposts are not significantly different. Glucose deficiency is compared for different traps in Figure 10G–I. The total area of quiescence and necrosis in created in the microwell is larger than in the other traps. According to these results, the flow generated in the U-shaped barrier created with microposts improves the distribution of glucose within the spheroid. The difference between the results of oxygen and glucose is due to the existence of oxygen permeable barrier that improves oxygen concentration distribution around the spheroid. Figure 10J shows maximum shear stress applied to the spheroid surface for the three traps. The maximum shear stress for the microwell is much lower than that for the U-shaped barrier. The existence of a gap in the U-shaped barrier increases the maximum shear stress slightly compared to those in the integrated counterpart.

### 5.8. Temporal Changes in Glucose Concentration without Supplying Medium

**Figure 11:**
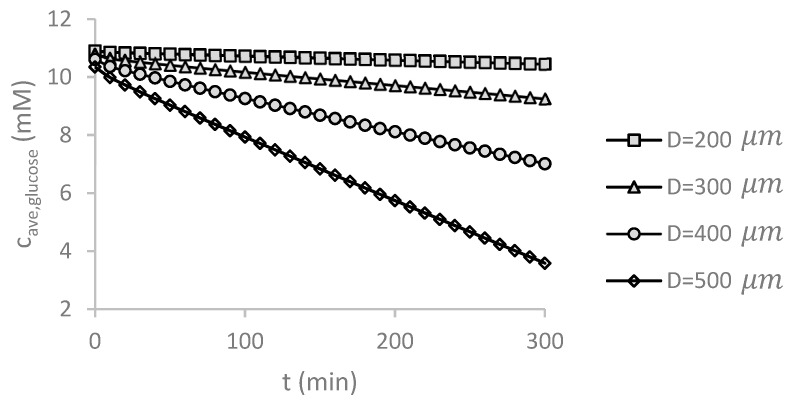
The average glucose concentration inside the spheroid changes over time for different spheroid diameter *D* without supplying the culture medium. For small spheroids, the temporal changes of inlet concentration of glucose are marginal. As the diameter of the spheroid increases, the cells consume more glucose over time and the continuous perfusion of the culture medium becomes essential to avoid necrosis. The simulations were carried out for $Q_{initial}=5\;\mu L/\min$ and $c_{0,glucose}=11\;\mathrm{mM}$.

The time-dependent solution for no medium flow was achieved for different spheroid diameters. The glucose concentration in all domains was obtained by solving the steady problem under $Q_{initial}=5\;\mu L/\min$ and $c_{0.\;glucose}=11\;\mathrm{mM}$. The average glucose concentration in the larger spheroids decreases significantly over time due to the consumption by the cells after suddenly stopping the fluid flow, Figure 11. Therefore, the medium flow cannot be stopped during the culture process, otherwise cells may suffer serious damage. For small spheroids, the rate of glucose consumption is so low and the glucose concentration in the medium and the spheroid remains in a favorable level for a long time. These results are close to those reported by Astolfi et al. [25], which concluded that continuous perfusion is not necessary for small spheroids.

**Figure 12:**
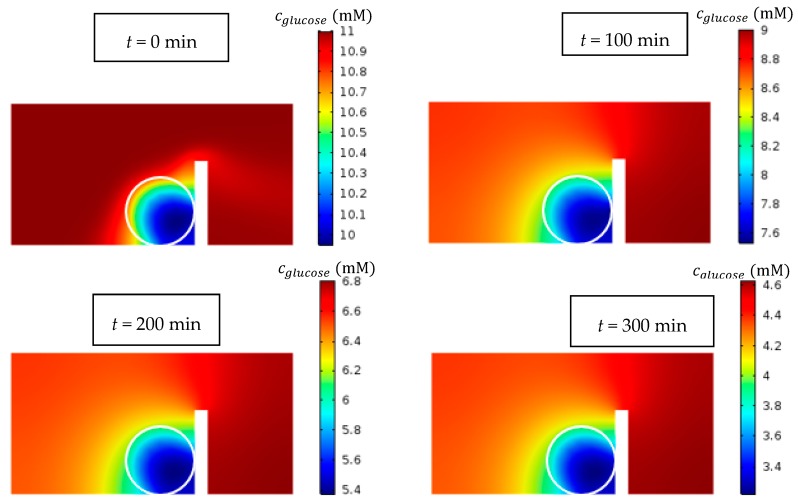
Glucose concentration contour at different times in the middle *x*–*z* plane inside and outside of the spheroid (color bar is in mM). Over the time, the possibility of necrosis increases. The simulations were carried out for $Q_{initial}=5\;\mu L/\min$, *D* = 500 μm and $c_{0.\;glucose}=11\;\mathrm{mM}$.

Figure 12 shows the contour of glucose concentration in the middle plane through the channel and the spheroid (*D* = 500 μm). Over time, the low concentration of glucose appears in some region and the possibility of necrosis increases as the time passes.

## 6. Limitations of the Simulation 

In this study, the oxygen and glucose concentration distributions were assumed to be independent from each other. One can consider the effects of oxygen-glucose coupling on the simulation results. Also, the oxygen and glucose consumption rates of the multicellular aggregates were modelled based on Michaelis–Menten reaction terms. Thus, depending on the cell type and heterogeneity of the cell clusters, each corresponding constant should be pre-determined precisely based on empirical values. Finally, the effect of tumor growth or multicellular spheroid growth was not considered here. The growth equations can be coupled with concentration equations of oxygen and glucose. As explained before, there is a discrete approach to model a multicellular aggregate. This approach can be used to model tumor growth considering cell interactions, which are ignored here due to the mentioned assumptions. The spheroid can be modeled as a porous medium to consider the effect of culture medium flow inside the aggregate. All of the above can help to improve the prediction of experimental results to increase the accuracy.

## 7. Conclusions

In an attempt to provide a predictable platform for in vitro assays, continuity, momentum and convection–diffusion equations were solved numerically in a microfluidic bioreactor with different cell traps and oxygen permeable walls. The effect of various parameters affecting such microfluidic system, which is suitable for culturing and formation of multicellular spheroids, were evaluated. The results showed that increasing spheroid diameter decreased the concentration of glucose and oxygen inside the spheroid due to the enhancement of oxygen consumption. Subsequently, increasing the medium volume flow rate increased the concentrations of both glucose and oxygen. The reduction of inlet concentration can affect the concentration distribution, and may even lead to quiescence or necrosis especially for large spheroid diameters. Therefore, the flow of culture medium cannot be stopped for a long period of `time. Accordingly, a continuous flow is more appropriate for this system if large spheroids are cultured. Increasing the spheroid diameter decreased inlet concentration and medium flow rate, and consequently, the hypoxic or quiescent and necrotic regions are expanded. The deviation of streamlines due to the U-shaped barrier decreased the amount of shear stress on the multicellular spheroids such that the maximum shear stress applied on a spheroid surface approaches 9 mPa. Oxygen and glucose concentrations inside the spheroid for a system with any of the U-shaped barriers (either the integrated U-shaped barrier or the one created with the microposts) are much higher than those in the microwell. The flow created in the gaps between the microposts increases the glucose concentration compared to that in the integrated counterpart. Due to the permeability of the U-shaped barriers, the concentration of oxygen is high and almost the same for both types of the U-shaped traps. The results of this study can serve as a guideline for a microbioreactor with better design and control the parameters affecting the formation and culture of multicellular spheroids within such microfluidic platforms.

## Figures and Tables

**Table 1:** Geometric parameters.

Parameters	Values (μm)	Parameters	Values (μm)
Width of the channel	1000	Length of the rectangular cubes in barrier	300
Height of the channel	1000	Position of the barrier (center of cylinder)	(0,500,1000)
Length of the channel	2000	Spheroid diameter (*D*)	200, 300, 400, 500
Inner radius of the barrier (half of a cylinder and two rectangular cubes)	300	Position of spheroid (center)	(*D*/2,500,1300 − *D*/2)
Thickness of the barrier	100	Thickness of the top PDMS layer	1000
Height of the barrier	600	-	-

**Table 2:** The simulation properties [25].

Parameters	Values	Descriptions
$\rho_{H_{2}O–37\;℃}$	993.3 kg/m^3^	Density of water at 37 °C
$\mu_{H_{2}O–37\;℃}$	0.000692 Pa·s	Viscosity of water at 37 °C
$K_{O_{2}–H_{2}O}$	2.6 × 10^−9^ m^2^/s	Diffusion coefficient of O_2_ through H_2_O
$K_{O_{2}–PDMS}$	3.4 × 10^−9^ m^2^/s	Diffusion coefficient of O_2_ through PDMS
$K_{O_{2}–cancerous\;tissue}$	1.83 × 10^−9^ m^2^/s	Diffusion coefficient of O_2_ through cancerous tissue
$K_{glucose–H_{2}O}$	9.27 × 10^−10^ m^2^/s	Diffusion coefficient of glucose through H_2_O
$K_{glucose–cancerous\;tissue}$	2.7 × 10^−10^ m^2^/s	Diffusion coefficient of glucose through cancerous tissue
$S_{O_{2}–PDMS\;vs.\;H_{2}O}$	6.8	Solubility of O_2_ in PDMS vs. H_2_O
$S_{O_{2}–cancerous\;tissue\;vs.\;H_{2}O}$	4.81	Solubility of O_2_ in cancerous tissue vs. H_2_O
$S_{glucose–cancerous\;tissue\;vs.\;H_{2}O}$	1	Solubility of glucose in cancerous tissue vs. H_2_O
$V_{max.\;O_{2}}$	0.0203 mM/s	Maximum reaction rate of O_2_
$V_{max.\;glucose}$	0.01076 mM/s	Maximum reaction rate of glucose
$K_{m.\;O_{2}}$	0.00463 mM	Michaelis constant of O_2_
$K_{m.\;glucose}$	0.04 mM	Michaelis constant of glucose
